# Comment différencier une pneumonie COVID-19 et un œdème aigu du poumon? À propos d’un cas

**DOI:** 10.11604/pamj.supp.2020.37.1.25947

**Published:** 2020-09-17

**Authors:** Hicham Faliouni, Zakaria Lahlafi, Mohammed Malki, Zouhair Lakhal

**Affiliations:** 1 Service de Cardiologie, Hôpital Militaire d'Instruction Mohammed V, Faculté de Médecine et de Pharmacie de Rabat, Université Mohammed V, Rabat, Maroc

**Keywords:** COVID-19, insuffisance cardiaque, pneumonie, COVID-19, heart failure, pneumonia

## Abstract

Nous rapportons le cas d´un patient âgé de 64 ans ayant comme antécédent une cardiopathie ischémique en dysfonction ventriculaire gauche en rapport avec des lésions tritronculaires relevant d´un traitement médical admis pour dyspnée. L'association de l´insuffisance cardiaque à la pneumonie COVID-19 est discutée. La distinction entre ces deux pathologies repose sur un faisceau d´arguments cliniques, biologiques et radiologiques.

## Abstract (en)

We report the case of a 64-year-old patient with a history of coronary artery disease and severe left ventricular dysfunction ischemic requiring medical therapy only admitted for dyspnea. The association of heart failure with COVID-19 pneumonia is discussed. The distinction between these two pathologies is based on a set of clinical, biological and radiological arguments.

## Introduction

En Décembre 2019, une épidémie de pneumonie causée par un nouveau coronarvirus (COVID-19) est apparue à Wuhan et s´est rapidement étendue à travers la Chine et les différents autres pays [1]. Le virus en cause, le SARS-CoV-2 (severe acute respiratory syndrome coronavirus 2) est doté d´une importante infectiosité même au cours de la phase asymptomatique et d´une virulence vraisemblablement sous-estimée. La manifestation la plus virulente de COVID-19 est le syndrome de détresse respiratoire aiguë (SDRA), des études préliminaires ont montré que certains patients peuvent développer des lésions cardiovasculaires graves [2]. L'insuffisance cardiaque est la maladie la plus fréquente rencontrée par les cardiologues. Les antécédents de contact et la manifestation clinique peuvent ne pas être clairs dans certaines circonstances. Sachant que de 30% des prélèvements des tests “polymerase chain reaction” (PCR) sont de faux négatifs lors de la présentation initiale [3]. La tomodensitométrie thoracique a été fortement recommandée pour le dépistage des patients suspectés de SARS-CoV-2 [4] car elle montre des caractéristiques radiologiques typiques chez presque tous les patients atteints de COVID-19, Nous montrons à travers une observation comment différencier une pneumonie COVID-19 et un œdème aigu du poumon.

## Patient et observation

**Figure 1:**
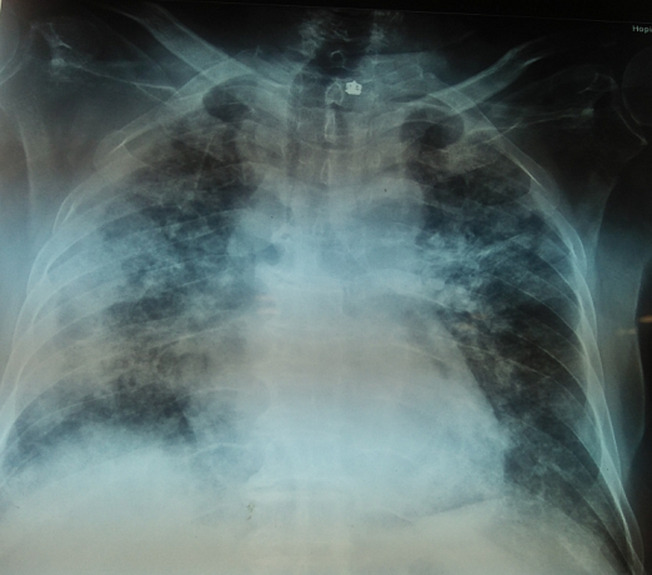
radiographie pulmonaire montrant une cardiomégalie avec un syndrome alvéolo-interstitiel bilatéral

Il s´agit d´un patient de 64 ans ayant comme facteurs de risque cardiovasculaires une hypertension artérielle sous bithérapie bien équilibrée et un diabète de type 2 de découverte récente et comme antécédent un syndrome coronarien aigu avec sus décalage du segment ST en antéroseptoapical il y a un mois compliqué d´une dysfonction systolique du ventricule gauche en rapport avec des lésions tritronculaires relevant d´un traitement médical. Il présente une dyspnée d´effort depuis 3 jours dans un contexte fébrile, motivant son hospitalisation. A l´examen clinique, le patient est dyspnéique au repos, apyrétique, avec une fréquence cardiaque à 65 battements par minute et une tension artérielle à 110/60 mmHg et une saturation artérielle en oxygène à l´air ambiant à 75%. A l´auscultation cardiaque, le rythme est régulier sans souffle. Il n´y a pas de râles crépitants ni ronflants. A l´électrocardiogramme, le rythme est régulier et sinusal à 70 battements par minute. Il existe des séquelles de nécrose en antéroseptoapical. La radiographie thoracique montre une cardiomégalie avec un syndrome alvéolo-interstitiel bilatéral (Figure 1). Le bilan biologique objective une hémoglobine à 11 g/dl, des leucocytes à 17 600/mm^3^ avec une lymphopénie à 800/mm^3^ sans thrombopénie, une CRP à 125 mg/l, une ferritine sérique à 707 ng/ml et enfin des LDH à 276 UI/l. Le dosage de la NT proBNP n´a pas été réalisé. La gazométrie artérielle a montré une hypoxie à 59 mmhg et une hypocapnie à 23 mmhg.

**Figure 2:**
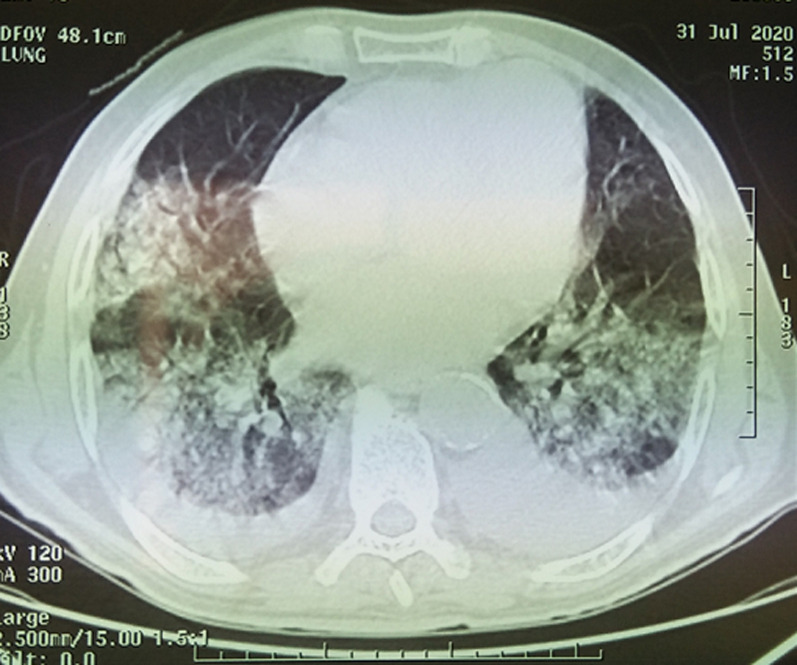
scanner thoracique montrant des images pulmonaires bilatérales en verre dépoli grade CORADS 5

Le ventricule gauche est non dilaté, siège de troubles de la cinétique segmentaire en dysfonction systolique avec une FEVG à 28%. Le ventricule droit est non dilaté de bonne fonction systolique. Les pressions de remplissage du ventricule gauche sont basses avec une pression artérielle pulmonaire systolique à 40 mmHg. Un test de PCR à la recherche du virus Covid-19 est négatif. Par contre, la tomodensitométrie (TDM) thoracique montre des images pulmonaires bilatérales en verre dépoli grade CORADS 5 (Figure 2). Devant la symptomatologie respiratoire, les données échocardiographiques, les images radiologiques très évocatrices de l´infection par la COVID-19, l´augmentation de la CRP et malgré la négativité de la PCR, le patient est traité par l´hydroxychloroquine et d´azithromycine en association avec l´oxygénothérapie et son traitement à visée cardiologique. Un deuxième test PCR à la recherche du virus COVID-19 a été prévu après 48 heures mais malheureusement le malade est décédé.

## Discussion

**COVID ou non COVID:** il s´agit d´un cas particulier d´un patient connu coronarien qui présente une dyspnée d´effort dans un contexte fébrile avec, au scanner thoracique, des lésions typiques d´infection COVID-19 grade CORADS 5. Dans une étude rétrospective [5] incluant des patients avec un diagnostic de pneumonie COVID-19 ou d'insuffisance cardiaque et qui ont subi une imagerie par tomodensitométrie. On constate qu'il existe certaines similitudes dans les caractéristiques d'imagerie pour les patients atteints d'insuffisance cardiaque et de pneumonie COVID-19. Les deux maladies avaient des opacités en verre dépoli, une consolidation, un motif de pavage fou et un épaississement septal, ce qui rendra difficile la différenciation de l'insuffisance cardiaque du COVID-19 par tomodensitométrie. Cependant, avec un contrôle minutieux de l'imagerie, le type de distribution des deux types de maladies était significativement différent. L'insuffisance cardiaque était plus susceptible d'avoir une distribution centrale et gravitationnelle, tandis que la COVID-19 avait généralement une distribution plus périphérique. Surtout, il y a plus de lésions de morphologie arrondie dans COVID-19 que dans l'insuffisance cardiaque. Dans le détail, bien que les deux maladies aient un épaississement septal, l'insuffisance cardiaque a généralement plus d'épaississement péribronchovasculaire et un épaississement septal interlobulaire voire fissural. Alors que COVID-19 affectait généralement le plus petit septum.

Il est intéressant de noter que les caractéristiques d'imagerie de scanner des deux maladies peuvent être mélangées chez un patient souffrant d'insuffisance cardiaque et de COVID-19. Malgré un test de PCR négatif, ce patient est considéré définitivement comme COVID-19 positif et traité comme tel, sachant que de 30% des prélèvements des tests PCR sont de faux négatifs et connaissant la haute valeur diagnostic du scanner. En effet, une étude chinoise [6] réalisée entre le 6 janvier et le 6 février 2020 montre que la sensibilité de la TDM est de 97%, sur la base des résultats positifs par RT-PCR, et de 75% sur les patients négatifs par RT-PCR. Ainsi, après l´analyse des données d´imagerie et de tests de laboratoire, il en ressort que la TDM thoracique est supérieure au test biologique dans le diagnostic de la COVID-19. Un prélèvement lors d´un lavement broncho-alvéolaire pour pouvoir retrouver l´ARN viral et étayer le diagnostic peut être intéressant dans ce cas.

## Conclusion

Dans le contexte épidémique actuel, l´infection au coronavirus devrait faire partie des diagnostics à envisager chez des patients présentant des signes respiratoires. Il est donc essentiel que les cardiologues se familiarisent avec les caractéristiques cliniques, biologiques et radiologiques qui différencient l'insuffisance cardiaque de la pneumonie COVID-19.
